# Cutting into the Mirror: Association of Body Image Concerns with Non-Suicidal Self-Injury in Adolescents and Young Adults with Eating Disorders

**DOI:** 10.3390/ejihpe15020023

**Published:** 2025-02-09

**Authors:** Francesco Maria Piarulli, Anna Margari, Francesco Margari, Emilia Matera, Giuseppina Viola, Claudia Maiorano, Gabriele De Agazio, Fabio Tarantino, Valeria Carruolo, Maria Giuseppina Petruzzelli

**Affiliations:** 1 Department of Translational Biomedicine and Neurosciences (DiBraiN), University of Bari “Aldo Moro”, 70124 Bari, Italy; piarullif@gmail.com (F.M.P.); francesco.margari@uniba.it (F.M.); viola.giusi@libero.it (G.V.); gab.deagazio@gmail.com (G.D.A.); tarantinofabio295@gmail.com (F.T.); valeriacarruolo@gmail.com (V.C.); maria.petruzzelli@uniba.it (M.G.P.); 2 Department of Mental Health, ASL Bari, 70124 Bari, Italy; clamaio91@gmail.com; 3 Interdisciplinary Department of Medicine, Section of Criminology and Forensic Psychiatry, University of Bari “Aldo Moro”, 70124 Bari, Italy; margarianna2@gmail.com; 4 Department of Precision and Regenerative Medicine and Ionian Area (DiMePRe-J), University of Bari “Aldo Moro”, 70124 Bari, Italy

**Keywords:** non-suicidal self-injury, eating disorders, body image concern, emotional dysregulation

## Abstract

Non-suicidal self-injury (NSSI) is a transdiagnostic behavior often found in patients with eating disorders (EDs). Both conditions plateau in adolescence and share psychopathological traits. Our study focuses on body image concerns, a complex psychopathological construct associated with both NSSI and ED, as a shared risk factor between the two. This study included 73 participants aged 14–24 recruited from the Eating Disorders Day Hospital, University Hospital of Bari, Italy, divided into two groups: those with an ED and NSSI (ED + NSSI) and those with an ED without NSSI (ED-only). Using standardized assessments such as clinical and demographical data, the Body Uneasiness Test (BUT-a), and the Eating Disorder Inventory (EDI-2), this study found that the ED + NSSI group exhibited significantly higher body image concerns in all main scales and subscales of BUT-a and EDI-2. Moreover, the ED + NSSI group presented higher scores on psychopathological traits associated with a more severe ED, namely Ineffectiveness, Social Insecurity, and Asceticism. Finally, patients in the ED + NSSI group were diagnosed with a higher degree of depressive disorders. These findings highlight significant associations between body image concerns and NSSI in patients with an ED, also showing a higher risk of psychiatric comorbidities and a more severe ED profile in these patients.

## 1. Introduction

Eating disorders (EDs) are serious psychiatric illnesses characterized by abnormal eating or weight-control behaviors and a preoccupation with food, body weight, or shape in distinct patterns for each diagnosis, with onset usually during adolescence. According to the most recent reclassifications of DSM-5-TR and ICD-11 (American Psychiatric Association, 2022; World Health Organization, 2019), EDs encompass anorexia nervosa (AN), bulimia nervosa (BN), binge eating disorder (BED), and other specified feeding and eating disorders (OSFED) as part of the more general chapter of “feeding and eating disorders” (FED) (Treasure et al., 2020).

EDs have a lifetime prevalence above 10% and a point prevalence of at least 5%, and rates are increasing in many parts of the world, with a decrease in the mean age of onset (Solmi et al., 2024). Individuals with an ED may present with severe disturbances in body image that play a central role in the initiation, persistence, and severity of ED symptomatology (Moccia et al., 2022).

Body image has been described as a broad multidimensional construct that encompasses the internalized view one has of one’s body, moving on a continuum from positive feelings of appreciation and enjoyment to negative feelings of loathing and distress (McLean & Paxton, 2019). Specific aspects of body image disturbance, such as body dissatisfaction, fear of weight gain, preoccupation and/or overvaluation of weight and shape, are recognized as being distinct from one another and having distinct relationships with core ED symptoms (Cuzzolaro et al., 2006; McLean & Paxton, 2019). There are several variables that may play a role in the development of body image problems and EDs, including low self-esteem, perfectionism, social (or media) pressure, social (or peer) comparison, and the existence of and exposure to a constant thin beauty ideal (González-Sánchez et al., 2024). On the other hand, body dissatisfaction during adolescence has also been associated with multiple negative health outcomes, such as depressive episodes; risky health behaviors such as smoking, drug use, and high-risk alcohol consumption; and, crucially, self-harm (House et al., 2023).

Non-suicidal self-harm (NSSI) is characterized by the voluntary infliction of pain and scarring to one’s body without the conscious intent to endanger one’s life, that is, without suicidal ideation (Favazza, 1998). Body dissatisfaction may represent a critical risk factor for self-harm, as the two conditions co-occur in the same clinical cohorts, such as in people with ED, and in the same period of life, such as adolescence and young adulthood (Dir et al., 2013; Matera et al., 2021).

While the relationships between body dissatisfaction, disordered eating, and NSSI remain largely unclear, there are some studies that suggest that body dissatisfaction may be a shared factor between disordered eating and NSSI, which should be considered for both understanding and investigating interventions for NSSI in patients with an ED (Hovrud et al., 2020; Muehlenkamp & Brausch, 2012; Tie et al., 2022).

Based on this theoretical framework, the present study aims to explore the differences in body image concerns between ED patients with and without NSSI to better understand the psychopathological profile of these groups. Considering that different functional profiles may explain why some individuals do and others do not develop and maintain harmful behaviors, we hypothesized that adolescents with ED and NSSI have higher levels of dysfunctional attitudes and feelings toward the body, as well as a different pattern of symptoms and psychological features associated with eating disorders. To our knowledge, a growing interest in the multidimensional aspects of body image in relation to NSSI and EDs has developed in recent years, and several new studies have begun exploring such dimensions. For instance, Meneguzzo and Todisco (2024) highlighted the role of body uneasiness in severe and enduring eating disorders, while Kirkpatrick et al. (2024) provided a systematic review of NSSI prevalence among individuals with an ED, emphasizing the transdiagnostic nature of these behaviors.

In light of these new perspectives, the objective of this research was to examine body experience in eating disorder patients with and without NSSI and to compare psychopathological features associated with EDs in both clinical groups to answer the following research questions:

1. Are patients with both an ED and NSSI burdened by worse body image concerns?
2. How do psychopathological traits related to the eating disorder differ between ED patients with and without NSSI?
3. Are there any differences in psychiatric comorbidities (e.g., anxiety, depression, personality disorders) between ED patients with and without NSSI?

We enrolled two different clinical subgroups of ED patients, with and without NNSI:

(a) To examine the differences in age and gender;
(b) To explore the differences in BMI, diagnosis of ED subtype, and associated psychiatric comorbidities;
(c) To study specific emotional and behavioral dimensions of body image able to differentiate ED patients with and without NNSI;
(d) To characterize general psychopathological traits associated with ED in both clinical groups.

## 2. Materials and Methods

### 2.1. Subjects

We enrolled adolescent and young adult patients referred to the Eating Disorders Day Hospital, University Hospital of Bari, Italy, between January 2018 and November 2023. We included patients with a primary diagnosis of an ED (anorexia nervosa, unspecified eating disorder, bulimia, binge eating disorder) according to criteria from the DSM-5-tr (American Psychiatric Association, 2022). We subsequently subdivided the subjects into two subgroups based on the concurrent presence of NSSI (ED + NSSI) or absence of NSSI (eating disorder only, ED-only) (American Psychiatric Association, 2022). With NSSI mostly being an adolescent and young adult phenomenon, we included patients aged between 14 and 24 years old in both groups. Both sexes were included in this study.

Exclusion criteria comprised the presence of intellectual disability, autism spectrum disorder, or schizophrenia spectrum disorder because of the potential interference of symptoms from these diseases with the accuracy of the data recollection, especially regarding self-report clinical scales. Other comorbid psychiatric diagnoses were not considered exclusion criteria, as they were investigated as part of the aims of this study. Lastly, adult patients who were unable to provide informed consent, as assessed by the research team, were excluded from this study (Table 1). For underaged patients, consent was provided by parents/legal guardians.

Psychiatric diagnoses (primary, secondary, and NSSI) were performed, according to DSM-5-tr (American Psychiatric Association, 2022) criteria, by an experienced psychiatrist based on the clinical symptoms and medical history collection. Standardized instruments such as the Eating Disorder Inventory (EDI-2) (Thiel et al., 2006) and the Ottawa Self-Injury Inventory (OSI) (Nixon & Cloutier, 2005) supported the diagnoses of EDs and NSSI, respectively. Participants were administered Italian versions of EDI-2 and OSI. All scores were carefully reviewed by bilingual (Italian- and English-speaking) physicians before data analysis, pending validation studies of official Italian versions of the scales. Internal consistency was assessed for each OSI subscale using Cronbach’s alpha, (past-month NSSI thoughts: 0.84; past-month NSSI behaviors: 0.84; past-six-month NSSI thoughts: 0.82; past-six-month NSSI behaviors: 0.80). The reported values indicate good reliability for the used subscales.

This study was conducted according to the guidelines of the Declaration of Helsinki and approved by the Ethics Committee of the Azienda Ospedaliero Universitaria Consorziale Policlinico of Bari (Resolution n. 1761, December 2019).

### 2.2. Clinical Assessment

All participants underwent the administration of the Body Uneasiness Test—side A (BUT-a) and Eating Disorder Inventory (EDI-2) (Clausen et al., 2011; Cuzzolaro et al., 2006; Thiel et al., 2006).

#### 2.2.1. Body Uneasiness Test—Side A

BUT-a is a self-administered test aimed at assessing problems regarding the individual’s body image. It consists of 34 items. Items are rated on a 6-point scale, from 0 (never) to 5 (always), with higher scores indicating greater impairment. Five factors regarding Body Uneasiness are evaluated:

- Weight phobia (WP);
- Body image concern (BIC);
- Compulsive self-monitoring (CSM);
- Avoidance behavior (A);
- Sense of detachment and depersonalization toward one’s own body (D).

In addition to the total score, the Global Severity Index (GSI) or overall mean score is calculated by summing the scores from the clinical scale (BUT-a) and dividing by their total number (34). The scale was originally developed and validated in Italian, French, and English (Cuzzolaro et al., 2006). The Italian version of it was administered. Internal consistency was assessed for each subscale using Cronbach’s alpha, (WP: 0.93; BIC: 0.92 CSM: 0.95 A: 0.94 D: 0.94, GSI: 0.92). The reported values indicate excellent reliability for all subscales.

#### 2.2.2. Eating Disorder Inventory

The Eating Disorder Inventory (EDI) is a self-administered psychodiagnostics test that includes a first set of 3 subscales specifically evaluating attitudes and behaviors related to eating, weight, and body shape (Drive for Thinness, Bulimia, and Body Dissatisfaction) and a second set of 7 subscales investigating general psychological traits (Ineffectiveness, Perfectionism, Interpersonal Distrust, Interoceptive Awareness, Fear of Maturity, Asceticism, Impulsivity, and Social Insecurity). Internal consistency was assessed for each subscale using Cronbach’s alpha, (Drive for Thinness: 0.87; Bulimia: 0.89; Body Dissatisfaction: 0.87; Ineffectiveness: 0.87; Perfectionism: 0.89; Interpersonal Distrust: 0.87; Interoceptive Awareness: 0.87; Fear of Maturity: 0.88; Asceticism: 0.87; Impulsivity: 0.87; Social Insecurity: 0.87). The reported values indicate strong reliability for all subscales.

- Drive for Thinness: assesses excessive concern for one’s body thinness, fear of weight gain, and obsession with dieting.
- Bulimia: identifies individuals suffering from bulimia nervosa, assessing the tendency to think and experience uncontrollable episodes of overeating, purging, and self-induced vomiting.
- Body Dissatisfaction: considered a major factor in the development of ED, measures the degree of dissatisfaction with the overall shape of one’s body and particularly vulnerable areas (thighs, hips, buttocks).
- Ineffectiveness: assesses feelings of sadness, worthlessness, inner emptiness, low self-esteem, and self-devaluation that are predisposing psychological factors for eating disorders.
- Perfectionism: measures the belief that “others” only accept very high standards of personal performance and always expect exceptional results.
- Interpersonal Distrust: evaluates general rejection or unavailability to establish and maintain close interactions and the failure to express one’s own feelings.
- Interoceptive Awareness: measures confusion and uncertainty in recognizing and responding to others’ emotional states; also identifies uncertainty in identifying feelings of hunger/satiety.
- Fear of Maturity: refers to the desire to retreat into the reassuring world of childhood, stemming from the inability to face the biological and psychological experiences involved in transitioning to adulthood.
- Asceticism: measures self-discipline, self-limitation, self-sacrifice, and control of the needs of one’s own body and is especially related to reduced need for physical and sexual interactions.
- Impulsivity: assesses tendencies to act and react impulsively, attraction to substance use, recklessness, and destructiveness in social and interpersonal relationships.
- Social Insecurity: identifies psychological experiences where social interactions appear difficult, anxiety-inducing, and are to be avoided because they do not bring existential benefits.

### 2.3. Statistical Analysis

We performed a descriptive analysis of frequency, mean, standard deviation (SD), range, and median values for demographical and clinical data. The Chi-squared test was used to check for differences in gender, ED diagnosis, and general psychiatric diagnoses between the two subgroups. After performing the Shapiro–Wilk test for normality, which indicated a significant deviation from a normal distribution for both groups, the non-parametric Mann–Whitney test was used to compare the values of the BUT-a and EDI-2 subscales, age, and BMI between the two subgroups. We performed a preliminary and post hoc power analysis to assess the ideal sample size and to evaluate the actual statistical power of our study with the real sample size to better understand the scope and validity of our findings. Additionally, an analysis of covariance (ANCOVA) was conducted to further assess the influence of possible influencing covariates (age and BMI) in the results. Data were processed using R software version 4.4.1 for MacOS (R Core Team, 2021). The level of significance was set at *p*-value < 0.05.

## 3. Results

### 3.1. Differences in Demographics and Clinical Features

A total of 73 adolescents and young adults with any diagnosis of an ED were enrolled; 31 patients were included in the group ED + NSSI and 42 in the group ED-only, without NSSI. No significant difference in age and gender was found between the two groups. The mean age was 17.83 (SD ± 2.90) for the ED-only group and 16.74 (SD ± 2.13) for the ED + NSSI group. Male/female distribution was as follows: 40/2 for the ED-only group and 27/4 for the ED + NSSI group.

The ED + NSSI group showed a significantly higher BMI compared to the ED-only group (ED 18.78 SD ± 3.12, ED + NSSI 23.45 SD ± 7.36, U = 277.5, *p* < 0.001). Consequently, additional analysis of covariance regarding diagnoses, BUT-a, and EDI-2 included the BMI as a covariate to exclude its influence as a possible confounding factor.

Differences in the diagnosis of EDs and other psychiatric comorbidities are reported in Table 2 and Table 3, respectively. Among different diagnoses of EDs, ED + NSSI subjects were more likely than ED-only to be diagnosed with AN (X^2^ = 7.138, *p* = 0.008); moreover, in the ED + NSSI group, we found a significantly higher presence of comorbid major depression (X^2^ = 4.525, *p* = 0.033).

### 3.2. Differences in Body Image Alteration

The Mann–Whitney U test was performed and the ED + NSSI group showed significantly higher values compared to the ED-only group in the BUT Global Severity Index (U = 144.5, *p* < 0.001) and in all BUT-a subscales (WP: U = 169, *p* < 0.001; BIC: U = 189.5, *p* < 0.001; CSM: U = 261.5, *p* = 0.005; A: U = 174.5, *p* < 0.001; D: U = 201.5, *p* < 0.001).

An analysis of covariance was conducted to examine the effect of NSSI on the BUT-a General Severity Index and all subscales after controlling for the influence of the covariates age and BMI. The results of the ANCOVA and the post hoc Tukey test confirmed the findings of the Mann–Whitney U test at the same level of significance for each item (pTukey and detailed Mann–Whitney U test results are detailed in Table 4).

### 3.3. Differences Psychopathological Features Associated with ED

The Mann–Whitney U test was performed and the ED + NSSI group showed significantly higher values, compared to the ED-only group in the scores for Bulimia (U = 323, *p* = 0.039), Body Dissatisfaction (U = 266.5, *p* = 0.004), Ineffectiveness (U = 248, *p* = 0.002), Perfectionism, (U = 315, *p* = 0.030), Asceticism (U = 300, *p* = 0.017), and Social Insecurity (U = 291, *p* = 0.012).

An analysis of covariance was conducted to examine the effect of NSSI on EDI-2 scores after controlling for the influence of the covariates Age and BMI. The results of the ANCOVA and the post hoc Tukey test confirmed the findings of the Mann–Whitney U test for Body Dissatisfaction (pTukey = 0.028), Ineffectiveness (pTukey = 0.010), Asceticism (pTukey = 0.040), and Social Insecurity (pTukey = 0.006). The scores for Bulimia and Perfectionism were non-significant in the post hoc Tukey analysis, indicating the possible influence of BMI as a confounding factor for these two factors. pTukey for all EDI-2 scores and detailed Mann–Whitney U test results are detailed in Table 5.

### 3.4. Power Analysis

The preliminary power analysis was conducted hypothesizing an alpha of 0.05, aiming at a power of 80% (beta = 0.2) to detect a difference between groups in the most standardized test used (EDI-2). We anticipated a mean for Group 1 of 11 (±4 standard deviation) and of 8 for Group 2, with a difference in means of 3 raw scale points based on the means of the items of EDI-2 (Thiel et al., 2006). The power calculation tool detected a minimum global sample size of 56 subjects, with a minimum of 28 subjects per group (Kane, 2024).

The post hoc power analysis, based on our sample and effect sizes, detected a power of 0.99.

## 4. Discussion

This study contributes to the existing literature by providing deeper information on the psychopathological profiles of adolescent and young patients diagnosed with ED and NSSI.

The main finding of this study was that patients diagnosed with ED and NSSI (ED + NSSI group), when compared with ED patients without NSSI (ED-only group), present a more complex clinical profile both regarding DSM diagnoses and the dimensional assessment focusing on body image and other psychopathological features associated with EDs, confirming our hypotheses and answering our research questions 1 and 3.

First, in the ED + NSSI group, we found a significantly higher score on the BUT, indicative of greater discomfort in the relationship with one’s body. It is known that body dissatisfaction and related experiences constitute a risk factor for the development of eating disorders and are key diagnostic criteria for AN and BN (McLean & Paxton, 2019). It is possible that the level of severity of the disturbance in one’s body image could be a clinical marker capable of distinguishing patients with an ED at greater risk of engaging in self-harm behaviors. In fact, recent findings suggest that adolescents with an ED and NSSI may exhibit unique clinical characteristics, including heightened body dissatisfaction and comorbid psychopathologies, which, according to some studies, may mediate this relationship (Sesso et al., 2024).

Moreover, body image refers to the multifaceted psychological experience of embodiment, which includes perceptions, feelings, and thoughts associated with the individual’s body size and physical appearance (McLean & Paxton, 2019). It is therefore necessary to have a clearer understanding of which components of body image impairment are most involved in specific clinical conditions

In this study, we found that all dimensions explored by BUT were significantly more compromised in the ED +NSSI group, with higher scores of anxiety about weight gain and body size, concerns related to physical appearance; avoidance behaviors related to body image; obsessive tendencies in monitoring body size, shape, or weight; and feelings of detachment and estrangement from the body. These findings align with our hypothesis that people with a diagnosis of ED + NSSI could have a more severe profile in all dimensions of body image than patients with ED-only without NSSI.

On the other hand, the differences in the scores obtained from the EDI also show significantly higher scores in the specific domain relating to body dissatisfaction in the ED + NSSI group. Other EDI domains that significantly distinguish patients with NSSI from those without NSSI are Asceticism, Social Insecurity, and Ineffectiveness. In all three subscales, the ED + NSSI group showed a more severe profile, with higher subscale scores.

The item “Ineffectiveness” is a valuable proxy measure for negative affect with symptoms such as sadness, worthlessness, inner emptiness, low self-esteem, and self-devaluation. This finding is coherent with a direct link between self-harm and negative affect (Anestis & Joiner, 2011; Cyders & Smith, 2007; Dir et al., 2013; Het et al., 2012; Yusoufzai et al., 2022), which may also explain the higher ratio of depressive disorders found in the ED + NSSI group. The coexistence of negative affectivity, low mood, self-harm, and body image alteration may mark a significant subtype of more severe NSSI patients (Adrian et al., 2011; Anestis & Joiner, 2011; Matera et al., 2021; Yusoufzai et al., 2022). This finding is in line with studies underlying the existence of latent subtypes of self-injurious urges among individuals with disordered eating and NSSI, further emphasizing the broad heterogeneity within this population (Moussaoui et al., 2024).

“Social Insecurity” points out difficulties in social situations, with anxiety and avoidance behavior. The higher ratio of Social Insecurity in a sample of patients with both an ED and NSSI hints at the possibility that body image may play a role in real or imagined social interactions, a finding in line with the well-known role of social influence in the pathogenesis of NSSI (Bentley et al., 2014; Brown & Witt, 2019; Cataldo et al., 2021; Schwartz-Mette & Lawrence, 2019; Spears, 2021; Zhou et al., 2024). In this case, too, the finding in Social Insecurity is paralleled by the higher number of diagnoses of anxiety disorders, a finding that may also be explained by stress response dysregulations often found in NSSI patients (Jiao et al., 2022; Juruena et al., 2020; Long et al., 2024; Piarulli et al., 2023; Turner et al., 2016; Yao et al., 2023).

Taking into consideration both items (Ineffectiveness and Social Insecurity) and the higher rates of depressive disorders, the results may be explained by several mechanisms. On one hand, NSSI could be engaged as a dysfunctional coping mechanism to handle depressive symptoms, as suggested by previous studies, and this could explain the higher ratio of diagnoses in the ED + NSSI group (Cawood & Huprich, 2012; Jiao et al., 2022; Midkiff et al., 2018). Moreover, as suggested by previous studies, NSSI could interfere and interact with the stress response, mediating changes in the HPA axis that could have a negative impact on all previous mechanisms, creating a dangerous positive feedback effect (Piarulli et al., 2023; Reichl et al., 2016, 2019). All findings must however be contextualized in light of the high rate of body image concerns and discomforts. With this being one of the stronger findings of this study, the importance of social factors and body image problems in characterizing the more severe clinical profile of the ED + NSSI group cannot be understated (Black et al., 2019; House et al., 2023; Moccia et al., 2022; Pérez et al., 2018).

A separate consideration must be made regarding the item “Asceticism”, which refers to a higher degree of compulsive control and “sacrifice” over one’s body and signals poor sexual health. Consistent with the other findings, the ED + NSSI group shows a significantly higher proportion of asceticism. This finding is striking since NSSI itself represents a damaging behavior over the body and a way to impact and exert control over body appearance (Black et al., 2019; Favazza, 1998; Klonsky et al., 2014), so this psychopathological feature could be key to understanding the relationship between body image, ED, and NSSI. On the other hand, poor sexual health is a severe burden on the global health of psychiatric patients (Hensel et al., 2016; Hope et al., 2022). Previous evidence highlights the poor sexual health of people with an ED (Pinheiro et al., 2010), while limited data are available about sexual health in NSSI. A handful of studies point out the existence of “sex as self-injury”, a troubling phenomenon highlighting the troubled sexual health of people who engage in NSSI (Fredlund et al., 2017). Other studies highlight the possibility that a significant part of ED patients who also engage in NSSI may have been victims of sexual abuse in the past (Cucchi et al., 2016; Juli et al., 2024). These findings call for more comprehensive assessments addressing sexual health in this troubled population. This finding hints at the possibility that people with an ED and NSSI could experience a poorer relationship with their bodies regarding sexual behavior and sexual health.

Considering these findings, it is possible to answer research question number 2 stating that the ED + NSSI group, compared to the ED-only group, shows a more severe clinical profile regarding psychopathological areas connected to the ED and a more severe general psychopathological profile. ED individuals who self-harm thus may experience greater psychological distress and more pronounced features connected to disordered eating, corroborating our study’s hypotheses.

### Limitations and Future Research

The first limitation of this study is intrinsic to the study design: the cross-sectional design allows us to obtain insight into the relationship between EDs, NSSI, and body image but restricts the possibility of establishing causality.

Another limitation regards the modest sample size of our study. Even though the power analysis yielded strong statistical power, a larger sample size and the inclusion of unaffected controls would increase the generalizability of our findings.

Moreover, while the clinical setting may be helpful in informing clinicians about the characteristics of patients, the generalizability of the findings is limited.

As suggested by previous research, future studies should comprise longitudinal data to explore the temporal dynamics of the relationship between EDs, NSSI, and body image, better establishing causality and investigating the efficacy of specific interventions (Meier et al., 2024), possibly targeted at specific latent subtypes within these populations (Moussaoui et al., 2024).

The sexual health of this troubled population should also be further studied, given the higher risk of both previous sexual traumas and sex as self-injury (Cucchi et al., 2016; Fredlund et al., 2017; Juli et al., 2024).

A final limitation regards the use of Italian versions of the scales. While the Italian version of the BUT is officially validated (Cuzzolaro et al., 2006), to date, no officially validated Italian version of the OSI and EDI-2 are available. While we opted to use a non-validated Italian translation of these scales, all scores were carefully reviewed by bilingual (Italian- and English-speaking) physicians before data analysis. This decision was further supported by previous studies that successfully used these scales in Italian settings (Brytek-Matera et al., 2017; Cotrufo et al., 1998; Matera et al., 2021; Segura-García et al., 2015; Serra et al., 2022) and by the lack of more suitable tools for assessing eating disorder symptomatology and non-suicidal self-injury in Italian populations.

## 5. Conclusions

While confirming previous findings regarding the role of body image on NSSI and negative affect, our findings expand on this concept, showing the relationship between NSSI, body image concern, and EDs. Moreover, the coexistence of the three conditions can be viewed and interpreted as a marker of gravity, with ED + NSSI patients showing a more severe clinical profile, both from the diagnostical and the psychopathological point of view, calling for targeted interventions (Kirkpatrick et al., 2024; Meier et al., 2024). This finding should inform clinicians: representing a more severe cohort, ED patients who also self-harm could need a more comprehensive therapeutic approach, focusing on body image and related psychological traits to mitigate self-harming behaviors (Pérez et al., 2018). In this line, evidence-based therapeutic approaches that focus on emotional dysregulation and cognitive restructuring of body image perceptions (Artoni et al., 2020; Hessler-Kaufmann et al., 2020; Tatham, 2011) may prove beneficial to this specific cohort of patients.

## Figures and Tables

**Table 1:** Inclusion and exclusion criteria.

Inclusion Criteria	Exclusion Criteria
Age between 14 and 24	Diagnosis of intellectual developmental disorder
Primary diagnosis of ED	Diagnosis of autism spectrum disorder
Both sexes	Diagnosis of schizophrenia spectrum disorder
	Inability to provide informed consent

**Table 2:** Eating disorders DSM-5-tr diagnosis between groups.

ED Diagnosis	Total	ED-Only Number	ED + NSSI Number	X^2^	*p*-Value (Uncorrected)	PHI Coefficient	X^2^ (Corrected)	*p*-Value (Corrected)
Anorexia Nervosa	38	28	10	8.461	0.004	−0.34	7.138	0.008 *
Binge Eating	3	2	1	0.107	0.744	−0.038	0	1
Bulimia	10	7	3	3.596	0.058	0.222	2.408	0.121
Unspecified Eating Disorder	24	11	13	2.004	0.157	−0.166	1.354	0.245

* Difference significant to the Chi-squared test.

**Table 3:** Comorbidities according to DSM-5-tr diagnoses between groups.

Comorbid DSM-5-tr Diagnoses	Total	ED-Only Number	ED + Nssi Number	X^2^	*p*-Value (Uncorrected)	PHI Coefficient	X^2^ (Corrected)	*p*-Value (Corrected)
Major depression	20	7	13	5.725	0.017	0.28	4.525	0.033 *
Personality disorders	8	2	6	3.892	0.049	0.231	2.541	0.111
Anxiety disorders	4	0	4	5.734	0.017	0.28	3.513	0.061
ADHD	1	0	1	1.374	0.241	0.137	0.024	0.878
Learning disorders	4	2	2	0.098	0.754	0.037	0	1
Bipolar disorder	1	0	1	1.374	0.241	0.137	0.024	0.878

* Difference significant to the Chi-squared test.

**Table 4:** pTukey and detailed results from the Mann–Whitney U test for BUT-a items between the two groups.

BUT-a	U	*p* (Mann–Whitney)	Rank-Biserial Correlation	SE Rank-Biserial Correlation	pTukey (ANCOVA)
Weight phobia (WP)	169.000	<0.001	−0.629	0.151	<0.001 *
Body image concern (BIC)	189.500	<0.001	−0.584	0.151	<0.001 *
Compulsive self-monitoring (CSM)	261.500	0.005	−0.427	0.151	0.005 *
Avoidance behavior (A)	174.500	<0.001	−0.617	0.151	<0.001 *
Sense of detachment and depersonalization toward one’s own body (D)	201.500	<0.001	−0.558	0.151	<0.001 *
Global Severity Index	144.500	<0.001	−0.683	0.151	<0.001 *

* Significant at multiple comparison levels (Tukey).

**Table 5:** pTukey and detailed results from the Mann–Whitney U test for EDI-2 items between the two groups.

EDI-2	U	*p* (Mann–Whitney)	Rank-Biserial Correlation	SE Rank-Biserial Correlation	pTukey (ANCOVA)
Drive for Thinness	372.500	0.176	−0.204	0.150	0.172
Bulimia	323.000	0.039	−0.310	0.150	0.932
Body Dissatisfaction	266.500	0.004	−0.431	0.150	0.033 *
Ineffectiveness	248.000	0.002	−0.470	0.150	0.012 *
Perfectionism	315.000	0.030	−0.327	0.150	0.128
Interpersonal Distrust	338.500	0.067	−0.277	0.150	0.178
Interceptive Awareness	373.500	0.183	−0.202	0.150	0.330
Fear of Maturity	404.000	0.367	−0.137	0.150	0.426
Asceticism	300.000	0.017	−0.359	0.150	0.046 *
Impulsivity	348.000	0.090	−0.256	0.150	0.088
Social Insecurity	291.000	0.012	−0.378	0.150	0.006 *

* Significant at multiple comparison levels (Tukey).

## Data Availability

The datasets presented in this article are not readily available because the data are part of an ongoing study. Requests to access the datasets should be directed to the corresponding author (EM).
